# Undiscovered Roles for Transthyretin: From a Transporter Protein to a New Therapeutic Target for Alzheimer’s Disease

**DOI:** 10.3390/ijms21062075

**Published:** 2020-03-18

**Authors:** Tiago Gião, Joana Saavedra, Ellen Cotrina, Jordi Quintana, Jordi Llop, Gemma Arsequell, Isabel Cardoso

**Affiliations:** 1 Instituto de Investigação e Inovação em Saúde, Universidade do Porto, 4200-135 Porto, Portugal; tiago.giao@ibmc.up.pt (T.G.); joana.saavedra@ibmc.up.pt (J.S.); 2 Institut de Química Avançada de Catalunya (I.Q.A.C.-C.S.I.C.), 08034 Barcelona, Spain; ellen.cotrina@iqac.csic.es (E.C.); gemma.arsequell@iqac.csic.es (G.A.); 3 Research Programme on Biomedical Informatics, Universitat Pompeu Fabra (UPF IMIM), 08003 Barcelona, Spain; jordiramon.quintana@upf.edu; 4 CIC biomaGUNE, Basque Research and Technology Alliance (BRTA), 20014 San Sebastian, Guipúzcoa, Spain; jllop@cicbiomagune.es; 5 Centro de Investigación Biomédica en Red Enfermedades respiratorias – CIBERES, 28029 Madrid, Spain

**Keywords:** transthyretin, transport protein, neuroprotection, blood–brain barrier, Alzheimer’s disease, TTR tetrameric stability, amyloid diseases, cerebrospinal fluid protein, angiogenesis

## Abstract

Transthyretin (TTR), an homotetrameric protein mainly synthesized by the liver and the choroid plexus, and secreted into the blood and the cerebrospinal fluid, respectively, has been specially acknowledged for its functions as a transporter protein of thyroxine and retinol (the latter through binding to the retinol-binding protein), in these fluids. Still, this protein has managed to stay in the spotlight as it has been assigned new and varied functions. In this review, we cover knowledge on novel TTR functions and the cellular pathways involved, spanning from neuroprotection to vascular events, while emphasizing its involvement in Alzheimer’s disease (AD). We describe details of TTR as an amyloid binding protein and discuss its interaction with the amyloid Aβ peptides, and the proposed mechanisms underlying TTR neuroprotection in AD. We also present the importance of translating advances in the knowledge of the TTR neuroprotective role into drug discovery strategies focused on TTR as a new target in AD therapeutics.

## 1. Introduction

Transthyretin (TTR), formerly called prealbumin as it migrates just in front of the albumin band, in electrophoresis, is a plasma protein secreted mainly by the liver and choroid plexus [1]. The name “transthyretin” discloses its dual physiological role as a carrier for both thyroid hormones [2] and retinol, the latter through the binding to retinol-binding protein (RBP) [3]. TTR is the key protein in familial amyloid polyneuropathy (FAP), a systemic amyloidosis characterized by the deposition of amyloid derived from mutated TTR, with a special involvement of the peripheral nervous system and firstly identified and described by the Portuguese neurologist Corino de Andrade [4]. Amyloidosis refers to a group of protein aggregation diseases, characterized by the extracellular deposition of those proteins in different organs, consequently forming insoluble aggregates called amyloids [5]. TTR is also acknowledged for its neuroprotective role in the central nervous system (CNS), such as in Alzheimer’s disease (AD), a form of localized amyloidosis affecting the brain, and the most common form of dementia. In AD, the main constituent of the amyloid deposits is the β-amyloid (Aβ) peptide, in senile plaques. Nevertheless, TTR involvement in neuroprotection is part of very recent knowledge, not fully established and constantly evolving. This review will focus on the role of TTR in AD and will address emerging functions of this protein.

## 2. Transthyretin as a Transporter Protein

### 2.1. Transthyretin Structure and Synthesis

The TTR protein is a 55 kDa homotetramer, consisting of four identical subunits of 127 amino acids each [6]. Regarding the secondary structure, each monomer consists of a small α-helix and eight antiparallel β-strands, which are arranged in two β-sheets (DAGH and CBEF strands) forming a β-barrel. With the twofold axis of symmetry, it can structurally be considered a dimer of dimers. The dimerization is achieved through the interaction of the strands H and F of each monomer. These dimers combine to form the tetramer through a connection between the AB loop of one monomer and the H strand of the other. At the interface of the dimers emerges a hydrophobic channel which can accommodate ligands such as thyroxine (T4) [7].

TTR is mainly synthesized by the liver [8] and by the epithelial cells of choroid plexus in the brain [9,10]. Depending on the age of the individual, the concentration of TTR in plasma varies between 20 and 40 mg/dL. In newborns, TTR levels in plasma are approximately half of those found in adults. Values increase with age and start to decline after about 50 years of age [11,12]. TTR has a biologic half-life of about 2–3 days, in humans. In rats, the major sites of TTR degradation are the liver, muscle, and skin. There is no evidence of TTR degradation in the nervous system [13].

In cerebrospinal fluid (CSF), TTR levels are considerably lower, ranging between 1.5 and 2.5 mg/dL [14]. In spite of the low TTR levels, TTR represents 20% of the total CSF proteins. Noteworthy, >90% of TTR in CSF is synthesized in the choroid plexus.

### 2.2. Endogenous Ligands of Transthyretin

Plasma retinol (vitamin A) is carried exclusively by the retinol binding protein (RBP), which in turn forms a complex with TTR, avoiding the loss of the low molecular mass RBP (21 kDa) by glomerular filtration in the kidneys [15,16]. TTR has four binding sites for RBP on the TTR surface. However, only one RBP molecule binds to TTR, due to the steric hindrance and the limiting concentration of this ligand [3,17]. TTR is also involved in the distribution of T4 to tissues, after its secretion into the blood [2,18]. Even though TTR contains two binding sites for T4 in the hydrophobic channel at the dimer–dimer interface, it usually carries only one ligand due to the existing negative cooperativity [19]. Of note, this interaction with T4 is independent of that with RBP [15]. TTR is responsible for transporting 80% of T4 in the brain as opposed to plasma, where TTR only carries 15%, making TTR in the plasma more sensitive to destabilization [20,21,22], as we will discuss in the next topics. Studies on the synthesis, secretion, and uptake of TTR by human placenta also suggest that TTR plays an important role in the delivery of maternal thyroid hormone to the developing fetus [23].

### 2.3. Mutations in Transthyretin and Association with Disease

TTR tetrameric structure and its stability are extremely important for TTR to exert its physiological functions. TTR instability, caused by mutations or by other factors, leads to dissociation of the tetramer into altered monomers [24] which are prone to aggregate and deposit as amyloid fibrils, leading to cell toxicity and death. Mutations in TTR are associated with hereditary forms of TTR amyloidosis and the most frequent TTR variant is V30M that causes ATTRV30M amyloidosis, also known as familial amyloid polyneuropathy (FAP). FAP is an autosomal dominant neurodegenerative disorder present in endemic regions in Portugal, Sweden, Spain, and Japan, the largest focus being in Northern Portugal. This pathology is characterized by the extracellular deposition of amyloid fibrils from mutated TTR in peripheral nerves [4,25]. Approximately 140 mutations in TTR with amyloidogenic potential have already been described (http://www.amyloidosismutations.com/mut-attr.php). The V30M TTR variant is the most common amyloidogenic form in the pathology, but other clinically aggressive mutants have been described, e.g., the L55P [26,27], and the V122I, which leads to cardiac amyloidosis [28]. Contrarily, T119M mutation is recognized for having a protective role against the disease [29,30]. Nevertheless, a non-hereditary form of TTR amyloidosis also exists, the senile systemic amyloidosis, and is mainly associated with cardiomyopathy in aged people, over 80 years old, and the deposits are composed of wild type (WT) protein [31]. Besides amyloidosis, TTR instability also leads to accelerated clearance, resulting in lower levels of the protein [32] which in turn prevents TTR from fully exerting its functions, namely its neuroprotection roles, that will be further detailed ahead in this review.

TTR stabilization strategies are a promising therapeutic approach in FAP [33,34]. Such stabilization can be achieved through the use of small-molecule compounds binding in the T4 central binding channel and stabilizing the native tetramer [35,36,37]. Analogous strategies have been also proposed as potential therapeutic strategies in AD, as we will further discuss.

Although higher levels of TTR are usually an indicator of a better protein nutritional status [38], increased TTR has also been associated with disease, as in preeclampsia, where aggregated TTR, extruded into the maternal circulation via placental extracellular vesicles, was also reported [39]. Higher levels of TTR are also detected in diabetes type II [40,41] and gestational diabetes [38], suggesting that TTR levels must be kept in balance within healthy intervals. Too high or too low levels of TTR may contribute to the development of pathological conditions.

## 3. The Neuroprotective Role of Transthyretin

AD is a degenerative brain disorder without effective treatment. Aβ peptide, a central agent in the development of AD, accumulates in the brain, and studies revealed that disturbance in Aβ brain metabolism is central to the pathogenesis of the disease. Blocking the synthesis of Aβ does not appear to be effective for reducing the brain Aβ levels, as expected. The importance of Aβ clearance in AD pathogenesis, especially in late-onset sporadic AD has thus been raised, and the understanding of Aβ clearance mechanisms have provided new therapeutic targets.

Clearance of Aβ from the brain occurs via active transport at the blood–brain barrier (BBB) and blood-cerebrospinal fluid barrier (BCSFB), in a process that is partially mediated by the low density lipoprotein receptor-related protein 1 (LRP1) [42], in addition to the removal of the peptide by several enzymes, mechanisms that are impaired in AD. For example, reduction of the levels of the Aβ degrading enzymes such as neprilysin, of its efflux receptor LRP1 and transporter P-glycoprotein (Pgp), are reported, while its brain influx transporter receptor for advanced glycation end products (RAGE) is increased [43].

TTR is known to bind Aβ and to facilitate its clearance from the brain, and next we discuss its involvement in AD neuroprotection.

### 3.1. Transthyretin in Alzheimer’s Disease—The Evidence

TTR has been reported as a neuroprotector in AD with several lines of evidence supporting this claim. Comparative analysis of TTR evidenced a significant decrease in the concentration of this protein not only in the CSF [44] but also in plasma [45,46,47] of AD patients, compared to age-matched healthy individuals. Additionally, TTR levels in the CSF are negatively correlated with disease severity and abundance of senile plaques [48,49], and TTR levels in plasma negatively correlate with disease stage [46].

Moreover, it was reported that AD/TTR-deficient mice have an increase in Aβ production and deposition compared to AD/hemizygous TTR littermates [50,51], whereas overexpressing human WT TTR in an AD mouse model decreases neuropathology and Aβ deposition [52].

#### 3.1.1. The Transthyretin/Aβ Interaction

In 1993, Wisniewski initially described that Aβ40 fibril formation was inhibited upon incubation with human CSF [53]. This fact could be associated with the sequestration of Aβ by extracellular proteins circulating in CSF such as Apolipoprotein E (ApoE) and Apolipoprotein J (ApoJ) [53,54,55]. Among several proteins determined as Aβ carriers, Schwarzman and colleagues concluded that TTR was the main protein able to sequester the peptide in the CSF. This was the first evidence implicating TTR in AD, specifically in Aβ transport and its clearance [56]. The authors proposed the hypothesis of sequestration as a possible explanation for the peptide aggregation and consequent progression of AD. This hypothesis suggested that certain extracellular proteins sequester normally produced Aβ, thereby preventing amyloid formation and its toxicity. Amyloid formation would occur when sequestration failed [56,57,58], which could be related either with an Aβ overproduction, a reduction in the levels of sequestering proteins, inability of those proteins to interact with the peptide, deficient clearance mechanisms, or a combination of all the events described above.

In vitro, the protective effect of TTR, namely its modulation in Aβ aggregation and toxicity, was also confirmed. Cultured brain vascular smooth muscle cells, previously isolated from dogs and AD patients were characterized by the presence of intracytoplasmic granules containing Aβ, which were induced by the presence of ApoE. TTR was able to rescue the cells from this accumulation, and positive Thioflavin-S (Th-S) staining, initially observed, was no longer detected [59].

In spite of the contradictory literature, Shirahama and colleagues and Stein and co-workers reported the presence of TTR within plaques [60,61]; as a consequence, an in vivo interaction between those molecules was considered and its biological relevance was studied. In fact, some transgenic animal models have been generated and the impact of TTR in Aβ aggregation and its toxicity determined. As an example, Link and colleagues produced a *Caenorhabditis elegans* highly expressing Aβ42 in muscle cells that triggered amyloid deposition positive for Th-S staining. To determine whether TTR expression would inhibit the peptide aggregation, double transgenic strains for Aβ and TTR were generated and results suggested a reduction in the number of positive Th-S deposits [62]. Similar studies have been performed with mouse models and the majority suggested and confirmed a protective role for TTR. Stein and Johnson evaluated gene expression profiles in hippocampus and cerebellum of 6-month-old AD transgenic mice Tg 2576, which overexpressed a mutant form of the amyloid precursor protein (APP) yielding high Aβ levels in the brain. Results indicated that, compared with age-matched controls, levels of TTR and other proteins important for certain survival pathways were increased, and this could explain the slow progression and lack of some important hallmarks of AD pathology that characterize this model [63]. However, controversy arose since TTR mRNA was never observed in the human hippocampus but only in the choroid plexus and the meninges of the brain. Nevertheless, chronic infusion of an antibody against TTR into the hippocampus of these mice resulted in increased Aβ levels and tau phosphorylation, neuronal loss, and apoptosis. The authors suggested that the soluble α-secretase cleaved APP fragment, sAPPα, increases the expression of protective genes, such as TTR, that in turn confer neuroprotection [61].

Regarding the characterization of the TTR/Aβ, Costa and colleagues demonstrated that TTR is able to interact with monomeric soluble Aβ, as well as oligomers and fibrils, with similar binding affinities. Besides neutralizing Aβ toxicity, TTR inhibited oligomerization and promoted fibril disruption [64,65]. Recent data suggest that TTR interferes with Aβ by redirecting oligomeric nuclei into non-amyloid aggregates [66], probably affecting both the clearance rate and the population of cytotoxic Aβ assemblies. Very recently, Ghadami and co-workers showed that TTR exerts its protective role by binding of TTR to the Aβ oligomers thus inhibiting primary and secondary nucleation processes, resulting in the inhibition of toxicity of Aβ oligomers and preventing fibril growth [67].

The effect of TTR mutations in TTR binding to Aβ has also been investigated. Schwarzman and co-workers synthesized approximately 40 recombinant amyloidogenic/non amyloidogenic mutated TTRs and, by applying several in vitro techniques, they were able to demonstrate that TTR variants bound differently to Aβ. TTR E42G and L55P, strong amyloidogenic variants, were the only ones that completely failed to bind the peptide [58]. Costa and co-workers also used amyloidogenic and non-amyloidogenic TTR variants and obtained the following profile for the strength of the interaction of TTR variants with soluble Aβ peptide: T1119M > WT > V30M ≥ Y78F > L55P, indicating that the higher the amyloidogenic potential of TTR, the weaker the interaction with the peptide [64]. Since the amyloidogenic potential of TTR correlates negatively with its tetrameric stability, those results also indicate that the lower the stability of TTR, the weaker the TTR/Aβ interaction. Furthermore, the L55P TTR variant also could not prevent Aβ toxicity in culture, confirming that one of the aims of the TTR/Aβ interaction is to prevent the noxious effects of the peptide [68]. These observations also imply that the TTR species needed for this interaction is the tetramer. In support of this idea, it has been shown that genetic stabilization of TTR through the presence of the T119M allele which renders a more stable tetramer, is associated with decreased risk of cerebrovascular disease and with increased life expectancy in the general population [69], further demonstrating the importance of the TTR tetramer in the protein biological activity.

However, in the context of TTR neuroprotection in AD, there is no consensus on the TTR species that provides the best effect. Whether TTR instability demonstrated in AD [46,70] is a negative consequence or is a protective mechanism to deal with the excess of Aβ remains unresolved. In fact, and in opposition to what was described above, some authors reported that a kinetically stable monomeric variant of TTR (M-TTR) binds strongly to Aβ and is stronger at avoiding its aggregation and toxicity than the TTR tetramer [71,72,73]. Moreover, M-TTR protected neuroblastoma cells and rat primary neurons against oligomeric Aβ induced toxicity to a greater extent than tetrameric TTR [74]. According to some data, the formation of Aβ fibrils is suppressed in the presence of sub-stoichiometric amounts of M-TTR. Nevertheless, sub-stoichiometric levels of M-TTR are not good aggregation inhibitors. Instead, they co-aggregate with Aβ to promote the formation of large, non-fibrillar, insoluble micron scale deposits. Based on fluorescence correlation spectroscopy measurements, it was found that M-TTR does not interact with monomeric Aβ. Two-color coincidence analysis of the fluorescence bursts of Aβ and M-TTR labeled with different fluorophores shows that M-TTR co-assembles with soluble Aβ aggregates and this appears to drive the co- aggregation into amorphous precipitates [75].

Data on the structural nature of the TTR/Aβ interaction was initially obtained from computer-assisted modelling [56]. The model predicts the existence of an Aβ binding domain on the surface of each TTR monomer. Other studies by the same group reported that residues 30-60, especially the 38-42 region of TTR are the key structure of the binding domain to Aβ [57,76]. Further studies identified TTR residues in the strands A and G, in or near the hydrophobic T4-binding site, as involved in TTR/Aβ interaction. The involvement of the EF helix and loop, which is highly solvent exposed and prone to conformational changes, was also detected [73,77]. Another study, using structural and computational approaches, has shown that binding of the Aβ peptide is likely to occur on the surface of the protein, and that the EF helix and the loop may play an important role in this interaction. Further, this study found that the Aβ(12–28) is the main recognition element of the Aβ interacting with TTR [78].

#### 3.1.2. Mechanisms Involved in Transthyretin Neuroprotection in Alzheimer’s Disease

In vitro and in vivo studies suggest a direct interaction between TTR and Aβ peptide, as already discussed, resulting in the inhibition of Aβ aggregation, fibril disruption or both, and thus, in this view, TTR is a carrier protein.

In the last past years, increased evidence indicates that oligomeric species of Aβ formed in the aggregation process are more toxic than mature fibrils [79]. Several reports have shown that such oligomeric intermediate might also be modulated by interactions with molecular chaperone proteins [80]. These molecular chaperones can be therapeutic agents targeting such toxic species and are an exciting therapeutic target in neurodegenerative diseases. The aggregation process is highly complex and for this reason, the elucidation of the mechanism by which Aβ aggregates is very challenging. In recent years, the scientific community has focused on kinetic approaches to identify the mechanisms at molecular level of these chaperoning effects, trying to identify the species targeted by chaperones and to elucidate the steps inhibited at microscopic level. Understanding the aggregation process is therefore one important step towards therapy and diagnosis of the disease. The molecular aggregation mechanisms generating amyloid fibrils can be divided into nucleation (i.e., fibril-forming, events that modify the total number of aggregates) and growth mechanisms (events that lead to an increase in aggregate mass). The events of nucleation can be further classified as primary and secondary processes through consideration of whether they depend on the aggregate population.

Using these kinetic analyses, Cohen and colleagues have shown that a molecular chaperone, a human BRICHOS domain, delayed toxicity in brain mouse tissue [80]. The Bri2 protein is produced in the central nervous system, in the same cells as the Aβ precursor protein, APP, and colocalizes with senile plaques, interacts with Aβ in neurons, and increased amounts of different Bri2 forms have been found in human AD brains [81,82]. Interestingly, other researchers have found that the BRICHOS domain from the Bri2 chaperone is BBB permeable, reaching the brain parenchyma after peripheral administration [83]. A single point mutation of Bri2 BRICHOS, the mutant R221E, forms stable monomers and selectively blocks a main source of toxic species during Aβ42 aggregation. By reprogramming an endogenous chaperone, these authors have increased the chaperone activity [84].

Several studies revealed that disturbance in Aβ metabolism in the brain is central to the pathogenesis of the disease. In 2016, Alemi and colleagues in a transwell system showed that Aβ brain efflux, at the BBB has the participation of TTR. Using the human cerebral microvascular endothelial cell line (hCMEC/D3) as the BBB model, authors showed that TTR added to the brain side, but not TTR added to the blood side, increased brain-to-blood permeability of hCMEC/D3 cells to Aβ. This suggests a direct interaction between TTR and Aβ peptide, probably transporting it to its receptor at the membrane. Moreover, the researchers demonstrated that TTR is capable of crossing BBB from the brain to the blood but not in the opposite direction, which indicates that the protein participates in the removal of the peptide from the brain, but cannot be responsible for its entry back [85].

Additionally, other mechanisms involving TTR in AD have been disclosed. TTR has been described as a cryptic protease with metallopeptidase activity [86]. In vitro, the proteolytically active form of TTR is able to cleave Aβ peptides as well as their aggregates. The resulting peptides show lower amyloidogenic potential than that of the full-length peptide [65,87]. Nevertheless, it is necessary to determine whether this proteolytic activity is relevant for neuroprotection in vivo. Although choroid plexus is commonly credited as the only source of TTR in the CNS, it has been reported, in recent years, that hippocampal and cortical neurons are able to produce the protein [88,89], although mostly corresponding to in vitro approaches. A neuroblastoma cell line overexpressing the APP intracellular domain (AICD), evidenced that TTR is epigenetically regulated by this fragment in the brain. As a result, there is an increase in TTR expression accompanied by a decrease in Aβ levels [89]. This discovery suggests possibilities for selective manipulation of TTR gene expression in neuronal cells. As authors discuss, dependence of amyloid clearance proteins on histone deacetylases (HDAC) and the ability HDAC inhibitors to upregulate their expression in the brain opens new avenues for developing preventive strategies in AD [90,91]. Additionally, it is reported that TTR binds to the CTFβ fragment, a product of AICD fragment, preventing γ-secretase from cleaving and releasing Aβ peptide. This binding reduces levels of Aβ secretion, thus suggesting that TTR regulates the metabolism of APP [92]. Another study described that the expression of TTR in the hippocampus, in primary murine hippocampal neurons, and a SH-SY5Y neuroblastoma cell line is significantly enhanced by the heat shock factor 1 (HSF1) [93].

#### 3.1.3. Importance of Transthyretin Stability in Alzheimer’s Disease

In AD, early alterations in TTR are observed. Subjects with mild cognitive impairment (MCI) present lower levels of the protein which continue to decrease with disease progression [46]. Although the underlying reason for this is not fully established, it has been hypothesized that it is a consequence of TTR tetrameric instability, suggesting that TTR is required to be in the tetramer form for an optimal binding to Aβ. This stability of the tetramer is important since TTR variants more prone to dissociate into altered monomers show a fast clearance, resulting in lower plasma concentration, in FAP [32]. A screening for TTR mutations in AD subjects was performed several years ago and the results failed to detect mutations in the TTR gene, suggesting that the influence in the sequestration of Aβ is not affected by TTR mutations [94]. Two other studies found an association of TTR variants in AD patients [95,96], including rare genetic variants in the Han Chinese population [95]. Nevertheless, the association is not yet established and further studies are needed to confirm if variability in the TTR gene does play a major role in AD. Destabilization of TTR may result from other events such as acidification of the medium, failure of the folding system, or interaction with various components such as metal ions and carbohydrates. Accordingly, plasma TTR from AD patients showed decreased ability to bind T4 [46] and decreased folded/monomeric ratios [70]. Thus, it is hypothesized that, in AD, TTR is destabilized and consequently is more quickly cleared, resulting in the reported lower concentration levels. The loss in TTR tetrameric structure decreases Aβ affinity and therefore does not allow TTR to exert its protective effect in AD. These lines of evidence also led to the hypothesis that TTR stabilization would restore its clearance and levels, and consequently, its ability to bind properly to Aβ and to protect in AD [68]. Finally, it is important to refer to the fact that TTR expression, in the choroid plexus and in the liver, is regulated by 17beta-estradiol [97,98]. It is reported that the age-adjusted AD incidence is higher in women compared with men, mainly in advanced ages and it has been suggested that estrogens may play a relevant role in this process [99], hence, estrogens decreased levels observed in AD women [46] may also contribute to the decline in TTR concentration, in AD.

### 3.2. Transthyretin as Therapeutic Target in Alzheimer’s Disease

TTR is the major Aβ binding protein in CSF, while albumin binds most of the Aβ in plasma. Plasma Aβ may represent a somatic pool of the peptide in dynamic equilibrium with its brain sources that may facilitate a net efflux from brain to plasma. The Alzheimer Management by Albumin Replacement (https://clinicaltrials.gov/ct2/show/NCT01561053) clinical trial was designed to evaluate the efficacy and safety of short-term plasma exchange followed by long-term plasmapheresis with infusion of human albumin. Results show the reduction in disease progression in patients with mild to moderate AD [100]. These results open a new avenue with albumin as disease-modifying AD therapeutic.

Concerning TTR, compounds such as iododiflunisal (IDIF), resveratrol, dinitrophenol, 2-[(3,5-dichlorophenyl)amino]benzoic acid, and 2-[(3,5-difluorophenyl)amino]benzoic acid, bind in the T4 binding channel and have been reported to behave as TTR tetrameric stabilizers, thus strengthening the TTR/Aβ interaction [68,101]. Hence, chemical stabilization of TTR has been proposed as a therapeutic avenue in AD. Interestingly, in the model put forward by Gimeno and co-workers, the stabilizer IDIF was capable of binding to TTR at the central pocket without affecting Aβ peptide binding [78].

In a revealing experiment, and using an AD transgenic mouse model with TTR genetic reduction (AD/TTR+/-), it was reported that IDIF administration resulted in decreased amyloid burden and total Aβ brain levels, and in the improved cognitive function of the animals [102]. Importantly, recent data showed that the TTR/IDIF complex exhibits improved BBB permeability, as compared to TTR and IDIF alone [103], providing higher Aβ sequestering capacity, and adding to the therapeutic potential of TTR in AD. In a different work, administration of resveratrol, to the AD/TTR+/- mouse model, also produced decreased brain Aβ burden and raised plasma TTR concentrations [104], confirming the stabilization hypothesis, whereas administration of resveratrol to a different AD mouse model, although not inducing decreased plaque burden, increased TTR protein concentration in the brain [105]. Nevertheless, resveratrol may protect via different mechanisms as it is reported that this polyphenol promotes intracellular degradation of Aβ via a mechanism that involves the proteasome, while not affecting the production of the peptide [106]. Cellular studies also indicate that TTR-assisted Aβ transport at the BBB and the liver can be improved by stabilizing TTR, namely with IDIF and resveratrol [70].

Very recently, it has been shown that AD mice with TTR genetic reduction present a thicker basement membrane [107], a feature characteristic of AD, and probably reflecting vascular alterations thought to occur early, and prior to Aβ deposition, during AD development. Given the current negative view on Aβ-based therapies, TTR stabilization may circumvent this, providing the opportunity for early treatment.

### 3.3. Other Neuroprotective Roles and Newly Discovered Functions of Transthyretin

#### 3.3.1. Transthyretin Protection in the Central and Peripheral Nervous Systems

Several reports attribute neuroprotective roles to TTR, in different contexts, both in the central and in the peripheral nervous systems. In TTR-knock-out (TTR-KO) mice, levels of the neuropeptide Y (NPY), known as an antidepressant neurotransmitter, which also acts on energy homeostasis by increasing white adipose tissue lipoprotein lipase, were shown to be increased [108]. TTR is also able to cleave amidated NPY [109], probably contributing to the increased NPY levels reported in TTR- TTR-KO [108].

Furthermore, it has also been described that TTR-KO mice present memory impairment compared with wild type animals [110], indicating that the absence of TTR accelerates cognitive deficits usually associated with aging.

In addition, it was demonstrated that TTR acts as an enhancer of nerve regeneration, following the observation that TTR-KO mice have decreased ability to regenerate from a sciatic crushed nerve [111]. Later, the same authors showed that the absence of TTR leads to impaired retrograde transport and decreased axonal growth, and also that the effect of TTR in neurite outgrowth and nerve regeneration is mediated by megalin-dependent internalization [112]. Interestingly, in vitro, the proteolytic activity of TTR is important for the capacity of TTR to promote neurite outgrowth [109].

A relationship between TTR and ischemia has also been established. Santos and co-workers proposed that in a compromised heat-shock response, CSF TTR contributes to control neuronal cell death, edema, and inflammation, influencing the survival of endangered neurons [113] via megalin [114].

The involvement of TTR against Aβ aggregation and toxicity in the context of Alzheimer’s disease is now well established and future efforts to exploit the role of TTR as therapeutic tool are expected.

#### 3.3.2. Transthyretin as a Gene Regulator

TTR was described as being involved in the regulation of insulin-like growth factor I (IGF-I) receptor in the hippocampus. TTR is able to increase IGF-I receptor levels, demonstrated in null and wild-type mice and in vitro-cultured cells [115]. Subsequently, it was found that TTR has a synergistic action with IGF-I over the IGF-I receptor, activating the Akt pathway, which is a specific IGF-I receptor signaling pathway [116].

The clearance of Aβ from the brain to the periphery through the BBB is mediated mainly by LRP1 [117], being also one of the main Aβ receptors in the liver [118]. Alemi and colleagues showed that, in mice brains and livers, LRP1 is decreased in TTR −/− compared to TTR +/+. In vivo, LRP1 expression was higher in hCMEC/D3 cells, a model for the BBB, and in a hepatoma cell line, HepG2, in the presence of TTR. These results suggest a possible role of TTR in the modulation of this receptor [85].

GABA receptors, GABA_A_-Rs and GABA_B_-Rs, are activated by GABA (γ-aminobutyric acid) and play an important role in brain development and synchronization of the neural network activity. A deficit in GABA_A_-Rs leads to a lack in neurotransmission and is involved in epilepsy, anxiety, depression, schizophrenia, and autism, since these receptors are located on synaptic and extrasynaptic membrane.

Very recently, Zhou and colleagues studied the interplay between TTR and the δ subunit of GABA_A_ receptors, and demonstrated that TTR interacts with δ-GABA_A_-Rs, regulating their expression and function [119]. In this work, a monomeric mutant of TTR failed to produce the same effect, highlighting that this newly discovered function is dependent on the presence of the native TTR tetramer.

#### 3.3.3. Transthyretin and Angiogenesis

Angiogenesis, the process by which new vessels are formed, is a highly regulated event involving degradation of the basement membrane of existent vessels, migration and proliferation of the endothelial cells (ECs), and maturation of the new vessels. In opposition to conditions as cancer in which angiogenic stimulus becomes excessive, in neurodegeneration the angiogenic signal is insufficient, causing EC dysfunction, vessel malformation or regression, or preventing revascularization [120]. The basement membrane of brain vessels is thicker in AD [121] and as it occurs before Aβ deposition, it is speculated that it functions as a physical barrier to Aβ clearance across the BBB.

Previous work has implicated TTR in angiogenesis, but the pathways involved are far from being unraveled. A study has compared the expression of angiopoietin-2 (Ang-2) and vascular endothelial growth factor (VEGF) receptors 1 and 2, that are recognized as pro-angiogenic genes, using WT TTR and the most common TTR mutation, V30M, in human umbilical vein endothelial cells (HUVECs). Authors showed that the V30M mutation downregulated the expression of the genes under study, as compared to WT TTR. Beside this, WT TTR promoted more cell migration and survival relative to the TTR mutant [122]. However, this study did not analyze the effect of WT TTR per se; instead, the work studied a possible toxic effect of the V30M variant, concluding that it impacts angiogenesis, by inducing apoptosis.

In a different study, Shao and colleagues found several key genes involved in angiogenesis modulated by TTR in human retinal microvascular endothelial cells (hRECs) cultured with TTR in natural and simulated diabetic retinopathy (DR) environments (hyperglycemia and hypoxia). The levels of expression of these genes varied according to the environment in which the cells were inserted. It was also observed that TTR led to the repression of cell growth, migration, and tube formation in a DR environment. Conversely, in low glucose conditions these features were enhanced by TTR [123]. Lately, the same authors also observed that TTR, in a DR environment, represses the formation of new vessels by inducing cell apoptosis through a GRP78-dependent pathway [124]. Taking into account that TTR may be increased in diabetes type II, this protein might have inhibitory functions under hyperglycemic conditions, from the early stage of DR [40,41].

TTR has also been consistently referred in tumor research as a differentially expressed gene or protein. However, the processes in which TTR is involved were not precisely addressed. Recently, it has been reported that TTR levels were highly increased in human serum of lung cancer patients. TTR concentration was also enhanced in the serum, bronchoalveolar lavage fluid, alveolar type II epithelial cells, and alveolar myeloid cells of the CCSPrtTA/(tetO)7-Stat3C lung tumor mouse model.

Blood vessels participate in the regulation of inflammatory and tumorigenic processes through controlling cell migration, vessel permeability, and cell infiltration into organs. TTR demonstrated a great influence on a wide spectrum of endothelial cell functions stimulating proliferation and tube formation. Endothelial cell permeability and migration was also enhanced in the presence of TTR, contributing to the suppression of T cell proliferation [125]. Thus, participation in angiogenesis may be an underlying mechanism of TTR action.

However, TTR angiogenic potential has never been tested in vivo, nor in brain-related processes or disorders. Results obtained using the AD triple transgenic mouse model (3xTg-AD) showed increased Aβ 42 isoform in the choroid plexus and reduction of TTR expression. Those animals also presented thickening of the epithelial basal membrane and greater collagen IV deposition in capillaries in choroid plexus [126]. Using the AβPPswe/PS1A246E AD mouse model [51], established in different TTR genetic backgrounds, the thickness of the basement membrane was investigated in brain microvessels by evaluation of the collagen IV contents. As results showed, AD mice carrying only one copy of the TTR gene and AD animals without TTR, presented a thicker basement membrane, a feature characteristic of AD, when compared to mice carrying both copies of the TTR gene [107]. As vascular alterations such as thickening of the basement membrane occur early in AD, and prior to Aβ deposition, during AD development, targeting TTR may provide the opportunity for early treatment.

## 4. Conclusions

TTR, involved in the transport of thyroid hormones and retinol, was proposed as a protective protein in AD in the mid-90s, when Schwarzman and colleagues described this protein as the major Aβ binding protein in CSF. Growing evidence also suggests a wider role for TTR in CNS neuroprotection, including in ischemia, regeneration, and memory.

AD, the most common form of dementia, predicted to affect over 100 million all over the world by 2050, is a neurodegenerative and irreversible disorder that affects the brain, and that slowly destroys memory and thinking skills. Drug discovery and development in AD is very challenging and although there are 132 new molecular entities in clinical trials, up to date there are no approved disease-modifying drugs for AD. New approaches are urgently needed.

The current view on AD regarding how Aβ should be tackled indicates that clearance of the peptide is a critical step to control its brain levels. Unfortunately, several of the Aβ clearance mechanisms are impaired in AD, such as reduced levels of its degrading enzymes and efflux receptors, and increased levels of its brain influx receptor. Restoring the levels of these proteins, namely of LRP1, is usually studied via gene delivery, which is complex and raises safety issues. Immunotherapy based strategies have also failed, so far.

**Figure 1:**
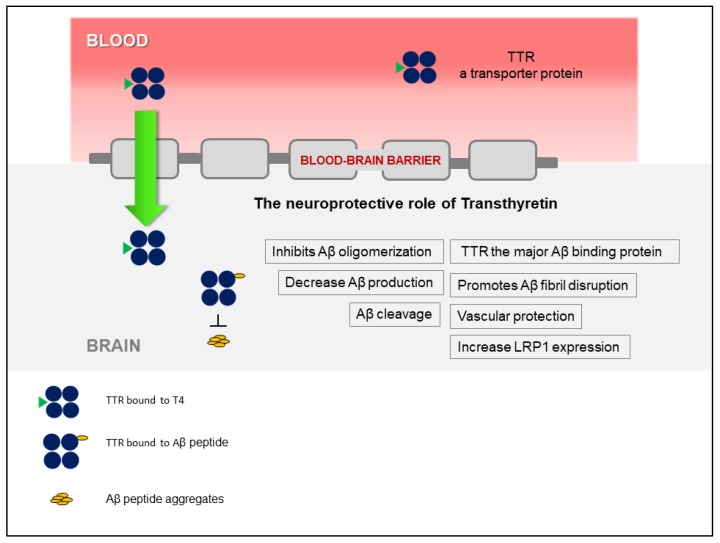
Schematic representation of the involvement of transthyretin (TTR) in neuroprotection in Alzheimer’s disease (AD), highlighting the different mechanisms involved.

The discovery that TTR stabilization, through the use of small-molecule compounds, enhances the TTR/Aβ interaction, opened a new avenue that relies on the recovery of TTR activity, without affecting gene expression. Figure 1 presents a schematic overview of the TTR neuroprotection in AD. Interestingly, TTR also regulates LRP1 levels, thus TTR promotes Aβ clearance at the BBB, directly, by assisting its elimination, and indirectly, by increasing the levels of the main Aβ efflux receptor, adding to the interest of using this protein as a therapeutic target in AD. However, the participation of TTR at the other brain barrier, the BCSFB, has not been investigated, yet. This and other studies to further dissect the underlying mechanisms of TTR neuroprotection and to design TTR-based AD therapeutic strategies, are still necessary.

In summary, this review presents the state of current research showing the neuroprotective role of TTR in AD together with its proposed mechanism of action. It also describes the TTR/Aβ complex as a possible new target for AD, and the recent research on molecules that enhance this protein–protein interaction. Additional pharmacological, drug discovery and structural biology research will be needed to validate TTR/Aβ as a target for AD, and to move active molecules addressing this target in preclinical assays towards clinical trials for AD patients.

## Abbreviations

TTR	Transthyretin
RBP	Retinol-binding protein
FAP	Familial amyloid polyneuropathy
AD	Alzheimer’s disease
CNS	Central nervous system
Aβ	β-amyloid peptide
T4	Thyroxine
CSF	Cerebrospinal fluid
WT	Wild type
Th-S	Thioflavin-S
APP	Amyloid precursor protein
ApoE	Apolipoprotein E
ApoJ	Apolipoprotein J
M-TTR	Monomeric variant of TTR
IDIF	Iododiflunisal
NPY	Neuropeptide Y
IGF-1	Insulin-like growth factor 1
LRP1	Low-density lipoprotein receptor-related protein 1
EC	Endothelial cell
BBB	Blood–brain barrier
VEGF	Vascular endothelial growth factor
HUVECs	Human umbilical vein endothelial cells
Ang-2	Angiopoietin 2
DR	Diabetic retinopathy
hRECs	Human retinal endothelial cells
